# Nutritional Risk, Micronutrient Status and Clinical Outcomes: A Prospective Observational Study in an Infectious Disease Clinic

**DOI:** 10.3390/nu8030124

**Published:** 2016-02-29

**Authors:** Oguzhan Sıtkı Dizdar, Osman Baspınar, Derya Kocer, Zehra Bestepe Dursun, Deniz Avcı, Cigdem Karakükcü, İlhami Çelik, Kursat Gundogan

**Affiliations:** 1 Department of Internal Medicine and Clinical Nutrition, Kayseri Training and Research Hospital, 38010 Kayseri, Turkey; osdizdar@gmail.com; 2 Department of Internal Medicine, Kayseri Training and Research Hospital, 38010 Kayseri, Turkey; osmanbaspinar1980@gmail.com (O.B.); denav38@gmail.com (D.A.); 3 Department of Biochemistry, Kayseri Training and Research Hospital, 38010 Kayseri, Turkey; ayder78@yahoo.com (D.K.); ckarakukcu@hotmail.com (C.K.); 4 Department of Clinic Microbiology and Infectious Disease, Kayseri Training and Research Hospital, 38010 Kayseri, Turkey; dr.zehrabestepe@hotmail.com (Z.B.D.); ilhamicelik@hotmail.com (İ.Ç.); 5 Division of Intensive care and Clinical Nutrition Unit, Erciyes University Medicine School, 38039 Kayseri, Turkey

**Keywords:** nutrition, micronutrients, infection, death

## Abstract

Malnutrition has been associated with increased morbidity and mortality. The objective of this study was to determine the nutritional status and micronutrient levels of hospitalized patients in an infectious disease clinic and investigate their association with adverse clinical outcomes. The nutritional status of the study participants was assessed using the Nutritional Risk Screening 2002 (NRS 2002) and micronutrient levels and routine biochemical parameters were tested within the first 24 h of the patient’s admission. The incidence of zinc, selenium, thiamine, vitamin B6, vitamin B12 deficiency were 66.7% (*n* = 40), 46.6% (*n* = 29), 39.7% (*n* = 27), 35.3% (*n* = 24), 14.1% (*n* = 9), respectively. Selenium levels were significantly higher in patients with urinary tract infections, but lower in soft tissue infections. Copper levels were significantly higher in patients with soft tissue infections. In the Cox regression models, lower albumin, higher serum lactate dehydrogenase levels and higher NRS-2002 scores were associated with increased death. Thiamine, selenium, zinc and vitamin B6 deficiencies but not chromium deficiencies are common in infectious disease clinics. New associations were found between micronutrient levels and infection type and their adverse clinical outcomes. Hypoalbuminemia and a high NRS-2002 score had the greatest accuracy in predicting death, systemic inflammatory response syndrome and sepsis on admission.

## 1. Introduction

Prevalence of malnutrition has been reported between 10% and 80% depending on the population, underlying diseases and test methods used [1,2,3]. Malnutrition has been associated with higher rates of complications, increased length of hospital stay and increased morbidity and mortality [4,5]. Malnutrition also increases the risk of infections and multiorgan dysfunction [6]. Thus, special attention should be paid to patients’ nutritional status, and proper nutritional support should be adopted in a timely manner to prevent adverse outcomes.

Nutritional status, dependent on both macro and micronutrients, is vital for human health. Different parameters are being developed to assess the nutritional status of hospitalized patients [7,8]. Nutritional risk screening (NRS)-2002 is recommended by the European Society of Parenteral and Enteral Nutrition for identifying patients at nutritional risk who may benefit from nutritional support in a hospital setting [5].

It is known that host nutritional status is one of the strong predictors of immunity [9]. Malnutrition is the most common cause of immunodeficiency worldwide and probably causes a significant percent of deaths from infectious diseases in developing countries [10]. A properly functioning immune system requires an adequate supply of micronutrients to both prevent damage of cells participating in the innate immune response and to restore tissues damaged from the host defense against the infectious agents [11,12,13]. Although the frequency of malnutrition risk in different patient population has been studied, the overall data on the nutritional status and micronutrient levels evaluated together in patients admitted to infectious disease departments has been very limited.

The objective of this study was to determine the nutritional status and micronutrient levels of hospitalized patients in an infectious disease department and to investigate whether their nutritional status and micronutrient levels were associated with adverse clinical outcomes.

## 2. Materials and Methods

### 2.1. Design and Participants

We conducted a prospective observational study in the department of infectious disease at Kayseri Training and Research Hospital, Turkey, which included all patients, aged 18 and above, who were admitted between May and October 2015. This study was performed in accordance with the Helsinki Declaration and approved by the Ethics Committee of Erciyes University Medical School (Ethics Committee approval code: 2015/266). We obtained written informed consent from all patients. The exclusion criteria were as follows: patients taking vitamin and trace microelements supplementation, patients that were hospitalized <72 h, patients who previously underwent bowel surgery, unconscious patients and patients whose answers were “No” to four questions in the initial screening of NRS-2002.

### 2.2. Data Collection

At the time of the patients’ enrollment, demographic information (age and sex) was recorded. Data on preexisting conditions including comorbidities, admission diagnosis, clinical characteristics, the condition that prompted admission, length of hospital stay (number of days between admission and discharge) and also destination post-hospital (home, transfer to intensive care unit (ICU) or death) were obtained from the clinical files. In order to understand the contribution of each variable on adverse clinical outcomes, we monitored and followed patients from admission to discharge from the hospital.

A blood sample was taken within the first 24 h of the patient’s admission for determination of serum selenium, vitamin B12, B6, thiamine, chromium, zinc, copper and ceruloplasmin levels. Serum ceruloplasmin levels were used for true determination of serum copper levels. Ceruloplasmin is an acute phase reactant. So, in an inflammation state, serum ceruloplasmin levels and serum copper levels were increased in correlation to increased ceruloplasmin levels. In addition, we measured the copper/zinc (Cu/Zn) ratio as an alternative biomarker assessing inflammatory and nutritional status and adverse clinical outcomes.

Systemic inflammatory response syndrome (SIRS) was defined as presence of two out of following four parameters: a temperature >38 °C, tachycardia (>90 pulse/min), tachypnea (>20/min) and leucocyte count >12,000 cells per mm^3^ or <4000 cells per mm^3^. Septic shock was defined as an infection-induced systemic inflammatory response with systolic blood pressure less than 90 mm Hg or a mean arterial pressure less than 70 mm Hg requiring the introduction of vasopressor drugs [14].

### 2.3. Assessment of Malnutrition Risk

The nutritional status of the study participants was assessed within the first 24 h following admission to the infectious disease department using the Nutritional Risk Screening 2002 (NRS 2002) [15]. Initial screening was made first. If the patient answered, “Yes” to any question, the screening in step 2 was performed. NRS 2002 step 2 screening combines two scores, the “nutritional score” ranging from 0 to 3 and the “severity of disease score” ranging from 0 to 3, plus one point if the patient is above 70 years of age. The NRS-2002 questionnaire was given to patients by physicians. The total NRS 2002 score (range 0–7) is the sum of the nutritional score, the disease severity score and the age adjustment. All patients that were hospitalized for six days or longer were reassessed every week and on the day of discharge using the same method as on admission.

Weight loss was evaluated using either the patient’s own history or previous medical records where available. Decreased food intake was assessed either subjectively by the patient’s history or objectively by nutritional diaries. The body weight was measured under fasting conditions in the morning after admission. The height was recorded from the case notes or by interviewing the patient.

All patients had a standard caloric intake (25–30 kcal/kg body weight/day) during hospital stay. Daily protein intake ranged from 1.0 to 1.2 g/kg body weight/day. Seventy percent of the remaining energy was provided by carbohydrates and 30% was provided by lipids. Because this study aimed to evaluate the levels of micronutrients in standard patients within the infectious disease clinic, nutrition support procedures conformed to clinical practice in our institution and were not specifically defined for this study.

### 2.4. Laboratory Analysis

On admission, a routine blood sample was taken and tested for complete blood count, routine biochemical parameters, acute phase reactants (erythrocyte sedimentation rate (ESR) and C-Reactive protein (CRP)), ceruloplasmin and vitamin B12. CRP levels were determined using nephelometric method with an autoanalyser (Siemens BN II, Erlangen, Germany) and ESR was analysed using westergren method with an autoanalyser (Alifax SPA, Padova, Italy). Serum and plasma samples were immediately centrifuged and frozen at −80 °C in aliquots for later measurement of thiamine, vitamin B6, selenium, zinc, copper and chromium.

A high performance liquid chromatography-based method was used to measure serum vitamin B12, B6, thiamine and chromium concentrations with the reference range of normality as 126 to 505 pg/mL, 4.1 to 43.7 ng/mL, 33 to 99 ng/mL and 0.7 to 28 µg/mL, respectively. Atomic absorption spectrophotometry was used to measure serum zinc, copper and selenium concentrations with the reference range of normality 70 to 150 µg/dL, 50 to 155 µg/dL, 46 to 143 µg/mL, respectively. In addition, we analyzed ceruloplasmin levels by nephelometry with the reference range of normality 0.2 to 0.6 g/L.

### 2.5. Statistical Analysis

Data are expressed as the mean ± standard deviation (SD) or the median (including the lower and upper quartiles). The normality and the homogeneity of the data were evaluated by Shapiro-Wilk test and Levene test, respectively. Comparisons between groups for continuous variables were performed using the Student *t* test (normal distribution) or the Mann-Whitney U test (non-normal distribution). Fisher test or the χ^2^ test was used for all categorical data. Pearson correlation was used to evaluate the association of continuous variables. Logistic regression analysis was used to determine the relative risks of developing SIRS and sepsis. Only the variables with a statistically significant association in the simple logistic regression model were included in the multiple logistic regression model. Odds ratios (OR) and 95% confidence intervals (CI) were determined. Cox regression models were used to predict hospital death. Kaplan-Meier survival analysis was used to analyze the frequency of death from high NRS-2002 score. All calculations used the SPSS statistical package (version 15.0; SPSS, Chicago, IL, USA). *p* < 0.05 was considered statistically significant.

## 3. Results

In this study, we evaluated 68 patients who were hospitalized in the infectious disease department, of which 37 (54.4%) were men and 31 (45.6%) were women, with a mean age of 62.8 ± 17.5 (23–100) years. The basic characteristics of the patients are shown in Table 1. The incidence of zinc, selenium, thiamine, vitamin B6, and vitamin B12 deficiency were 66.7% (*n* = 40), 46.6% (*n* = 29), 39.7% (*n* = 27), 35.3% (*n* = 24), 14.1% (*n* = 9), respectively. Twenty-two patients (32%) had multiple micronutrient deficiencies. Chromium levels were in the normal range for all patients. Copper levels were within the normal range or higher than the normal range. Mean ceruloplasmin level was 0.89 ± 4.4, which was higher than the normal range.

We investigated the effects of micronutrients and other nutritional parameters on the development of SIRS, sepsis and death (Table 2). Patients with SIRS had a higher NRS-2002 score, lower albumin, thiamine and zinc levels. Patients who had sepsis had a higher NRS-2002 score and chromium levels and lower albumin and thiamine levels. Logistic regression analysis was used to determine the relative risks of developing SIRS and sepsis. Only the variables with a statistically significant association in the simple logistic regression model were included in the multiple logistic regression model. In multiple logistic regression analysis, significant risk factors associated with SIRS were as follows: lower albumin, higher NRS-2002 scores and glucose levels (glucose levels of patients with SIRS or not: 188 ± 110; 119 ± 50, respectively) on admission (Table 3). Furthermore, multiple logistic regression analyses identified the following factors as independent risk factors for sepsis: lower albumin and NRS-2002 score on admission (Table 4).

**Figure 1:**
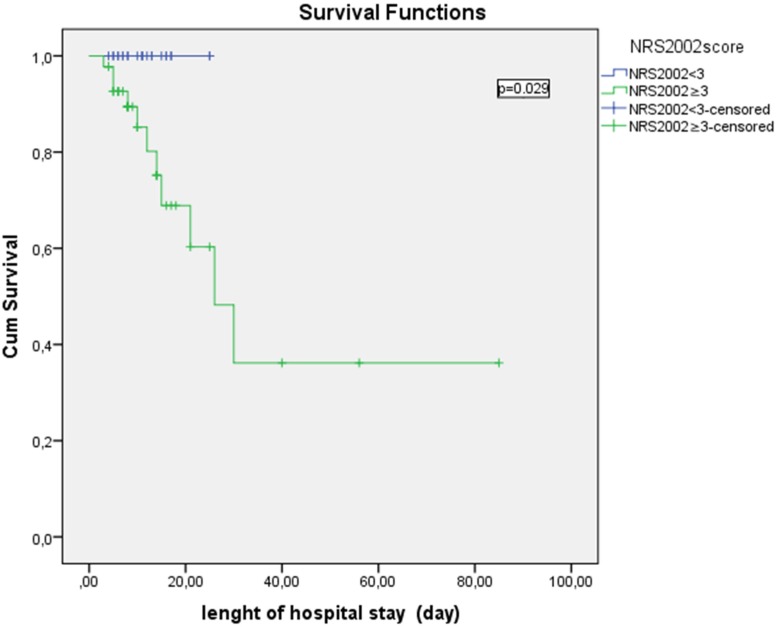
Kaplan-Meier Plots Showing Survival of Patients. Kaplan-Meier survival analysis demonstrated a significantly longer hospital survival in patients with Nutritional Risk Screening 2002 (NRS 2002) score <3 compared with patients who had NRS 2002 score ≥3 on admission. Patients were censored at discharge for this analysis.

Factors that were significantly associated with death from any infection included higher NRS-2002 scores, higher serum chromium levels and lower albumin levels. In the Cox regression models, lower albumin and higher serum lactate dehydrogenase (LDH) levels (LDH levels of expired patients or living patients: 628 ± 861; 299 ± 298, respectively) and NRS-2002 score were associated with an increased incidence of death in these patients (Table 5). In 68 patients who were hospitalized in the infectious disease department, Kaplan-Meier plots showed that there was a significant difference in survival between patients who had NRS-2002 score ≥3 or <3 on admission (Figure 1).

The serum thiamine levels showed a positive correlation with vitamin B6 levels (*r* = 0.508; *p* < 0.001), zinc levels (*r* = 0.442, *p* < 0.001) and copper levels (*r* = 0.267, *p* = 0.041). There was no correlation between other micronutrients levels. NRS-2002 scores on admission were negatively correlated with thiamine (*r* = −0.245, *p* = 0.044), zinc (*r* = −0.318, *p* = 0.013) and copper levels (*r* = −0.286, *p* = 0.028). The age of patients was negatively correlated with serum copper levels (*r* = −307, *p* = 0.018). In addition, serum micronutrients levels measured in the present study were not significantly correlated with length of hospital stay or BMI.

Considering the relationship between micronutrient levels and laboratory findings, there were positive correlations between serum chromium levels and urea, serum chromium and creatinine levels and serum chromium and leucocyte count (*r* = 0.264, *p* = 0.029; *r* = 0.241, *p* = 0.048; *r* = 0.269, *p* = 0.026; respectively) and between serum copper levels and ESR, serum copper and lactate dehydrogenase and serum copper and alkaline phosphatase levels (*r* = 0.357, *p* = 0.005; *r* = 0.300, *p* = 0.023; *r* = 0.377, *p* = 0.004; respectively). CRP and albumin levels did not show any correlation with serum micronutrients levels. However, CRP and ESR levels on admission were positively correlated with Cu/Zn ratio (*r* = 0.287, *p* = 0.027; *r* = 0.310, *p* = 0.017; respectively).

Selenium levels were significantly higher in patients with urinary tract infections (selenium levels of patients with and without urinary tract infection: 60.1 ± 13; 47.5 ± 13.4, respectively) (*p* = 0.001), but selenium levels were significantly lower in soft tissue infections (selenium levels of patients with or without soft tissue infection: 43.8 ± 8.7; 52.8 ± 14.4, respectively) (*p* = 0.010). Copper levels were significantly higher in patients with soft tissue infection (copper levels of patients with or without soft tissue infection: 169.2 ± 46.3; 137 ± 39.6) (*p* = 0.033). There was no significant association between other serum micronutrients levels and infection type. In addition, micronutrient deficiency did not increase the risk of development of mixed infections. Four patients were ongoing a hemodialysis program. Two of them had zinc, thiamine and vitamin B6 deficiency, one of them had vitamin B12 and vitamin B6 deficiency and one of them had a zinc deficiency only. Nine patients (13.2%) experienced a complication during the hospital stay, four had nephrotoxicity, two had thrombophlebitis, one had anaphylaxis, two had thrombocytopenia and pancytopenia. Ten patients (14.7%) had secondary infections during the hospital stay along with pneumonia in three patients and urinary infection in two patients. Patients who had secondary infection had significantly lower selenium levels (*p* = 0.029).

## 4. Discussion

Malnutrition upon hospital admission is associated with higher rates of infection and increased morbidity and mortality, which can be prevented if attention is paid to the nutritional care of patients [16]. Therefore, it is now widely recommended that hospitalized patients should be routinely screened for malnutrition. However, micronutrients are often ignored during nutritional screenings in practice. Nutritional status or micronutrient levels could be related to the probability of progressing from infection to overt/severe forms of disease (SIRS, sepsis) in patients. Thus, in the present study, we measured serum micronutrient levels in addition to nutritional screening with NRS 2002 and we mainly focused on evaluating the association of micronutrient levels and nutritional parameters with adverse clinical outcomes of patients who were hospitalized within the infectious disease clinic. Screening of micronutrient levels can help to identify or predict the risk of developing a condition and the features associated with micronutrient deficiency including complications and death.

Considering all clinic and laboratory parameters in multiple logistic regression analysis, low serum albumin and high NRS-2002 scores had the greatest accuracy in predicting death, SIRS and sepsis in patients admitted to the infectious disease clinic. NRS-2002 and serum albumin levels are parameters related to nutrition status. Therefore, in our study as in others, malnutrition was associated with increased risk of SIRS, sepsis and mortality and thus, poor clinical outcome and we can say that the nutritional status is more important than previously believed in the infectious disease department based on our results [17].

The association between high NRS-2002 score and mortality had been demonstrated in different studies, as in our study [18]. Furthermore, we showed a significant difference in survival between patients who had a NRS-2002 score ≥3 or <3. This result revealed the importance of the clinical use of NRS-2002 on hospital admission. Because of the long half-life and the interaction with inflammatory disorders [19], there is no consensus on the validity of the use of serum albumin as a parameter for nutritional diagnosis [20]. Despite that, low serum albumin levels were associated with an increase in hospital complications, length of hospitalization, and hospital mortality [21,22,23]. However, establishing a relationship between malnutrition and hospital death may be a difficult task due to the enormous range of factors that contribute to such outcomes. The presence of infection, also influenced by malnutrition, may itself be a cause of hospital mortality.

High LDH levels were another independent risk factor for mortality in our study besides nutritional parameters. Similarly, previous studies found LDH to be an independent predictor of hospital mortality [24]. We found hyperglycemia as an independent risk factor for SIRS but not sepsis. This result constitutes a contradictory situation. Regardless, the effects of hyperglycemia on infectious disease outcomes are highly controversial in literature and more studies are required.

Several factors could contribute to decreased levels of micronutrients in patients who had an infection including decreased appetite, decreased absorption due to diarrhea, or an increased requirement for nutrients for immune functions or tissue repair. In septic shock patients, much research has been directed towards selenium and thiamine, but other micronutrients have been largely ignored. In addition, there are limited studies investigating the relationship between micronutrient levels and infection type. The present study offers detailed information on this topic. It is not clear from the scientific literature if multiple micronutrient deficiency is a major problem in infectious disease clinic. We did not determine a significant relationship between multiple micronutrient deficiencies and adverse clinical outcomes such as sepsis and mortality. So, the clinical significance of multiple micronutrient deficiencies is obscure and further study is needed.

Thiamine plays an important role in glucose metabolism and thiamine deficiency leads to anaerobic metabolism and lactate formation. In our study, in patients with SIRS and sepsis, thiamine levels were significantly reduced and the frequency of thiamine deficiency was 46.4% and 55.6%, respectively. In two recent studies involving septic shock patients, the incidences of thiamine deficiency were 20% and 71.3% [25,26]. This discrepancy between frequencies of thiamine deficiency in sepsis could be explained by differences in illness severity or the patient population. At this point, more studies are needed to see the effect of thiamine deficiency on the course of sepsis and it is not practical to recommend routine thiamine evaluation in sepsis patients. The serum thiamine levels positively correlated with vitamin B6, zinc and copper levels were inversely correlated with the NRS-2002 score. This finding showed the need to think of nutrition as a whole.

Because of the relationship between lactate levels and mortality in critically ill patients, the association between thiamine deficiency and mortality has been explored with controversial results. In a retrospective study, Cruickshank *et al*. reported that thiamine deficiency was associated with higher mortality in ICU patients [27]. However, other studies have failed to demonstrate this association [28]. In our study, serum thiamine levels were reduced in patients who died, but this deficiency was not significantly associated with mortality.

Selenium is essential for the antioxidant status. Selenium deficiency is aggravated by acute illness [29]. So, in patients with severe sepsis or SIRS, there is a decrease in plasma selenium concentrations that could be associated with a decrease of antioxidant defenses [29]. In addition, Manzanares *et al.* showed that lower levels of selenium after ICU admission was a predictor of systemic inflammatory response syndrome [30] and Sakr *et al.* showed that in critically ill surgical patients, plasma selenium concentrations were generally low and were associated with greater tissue damage and increased ICU mortality [31]. In Europe, the general population had suboptimal selenium status [32]. However, there is no study on this subject from our country, Turkey. The frequency of selenium deficiency in the present study was 50% in patients with sepsis and was lower than other studies from Europe. We can relate this situation to soil structure and eating habits in our country. Furthermore, our results in sepsis patients do not agree with previous findings investigating the relationship between selenium levels and adverse clinical outcomes. We did not observe any increase of the length of hospital stay, any effect on developing any complication (such as organ failure) and any increase in mortality. But secondary infections during hospital stay were significantly higher in patients with selenium deficiency. We found that selenium levels were significantly higher in patients with urinary tract infections and significantly lower in soft tissue infections. There was no information about this relationship in literature. Further investigation is needed.

Zinc is essential in nearly every step of immune function and antioxidation [33]. Zinc deficiency is common among populations at high risk for sepsis mortality, including elderly, alcoholic, and hospitalized patients. Zinc deficiency leads to susceptibility to infections and has also been associated with decreased resistance to viral infection [34]. Abnormal zinc loss is seen in patients with diarrhea and without oral intake [35]. Because diarrhea is a common in the infectious disease department, the measurement of zinc levels should not be neglected.

In Mertens K *et al.* study, zinc and selenium concentrations were reduced in critically ill patients with increased oxidative stress and inflammatory biomarkers, particularly in patients with sepsis [36]. In our study, zinc levels were significantly lower in SIRS patients. However, although zinc levels were lower in expired patients, we did not demonstrate any relationship between zinc deficiency and mortality. Zinc supplementation has also been found to reduce mortality from pneumonia [37] and to be beneficial in preventing respiratory infection in some studies [38]. Although zinc levels were lower in our patients with pneumonia, there was no association between zinc deficiency and pneumonia.

Copper is involved in wound healing (essential for the synthesis of collagen and elastin), immune function and antioxidant defenses (copper-zinc superoxide dismutase) [39]. Songchitsomboon *et al.* found significantly increased serum copper concentrations in patients with infectious diseases [40]. Similarly, copper levels were within the normal range or increased in our study. Assessment of the status of copper levels is difficult as infection and inflammation increases ceruloplasmin levels. Serum copper levels were significantly higher in soft tissue infection. This association could be explained with the importance of copper in collagen tissue repair. Lee *et al.* found that serum concentrations of copper increased significantly several months following recovery from acute pulmonary exacerbation in patients with cystic fibrosis [41]. We did not find any association between copper levels and pulmonary infection and mortality.

Cu/Zn ratio proved to be a better predictor of disease severity and/or mortality than copper levels [42]. However, we did not show any association between Cu/Zn ratio and adverse clinical outcomes. Only a weak correlation was present between Cu/Zn ratio and CRP and ESR. Similarly, in Malavolta *et al.*’s study, Cu/Zn ratio was associated with all the inflammatory markers (CRP, ESR, IL-6) [42]. So, our results supported Malavolta *et al.*’s theory for Cu/Zn ratio as an important clinical inflammatory biomarker.

Chromium has been known to affect the immune response by influencing T and B lymphocytes, antigen-presenting cells and cytokine production. Until this study, the major condition related to its deficiency is impairment of glucose metabolism. To our knowledge, this is the first study to investigate the association between chromium levels and sepsis and death in the infectious disease department. Chromium levels were in the normal range in all our patients. But interestingly, sepsis patients and expired patients had significantly higher chromium levels. The clinical importance of this finding was obscure and further investigation is needed. High doses chromium supplementation or exposure to chromium from the environment has been reported to cause many adverse health effects [43,44]. For example, they inhibit many cellular processes and mutate genes that are critical to the immune response. Therefore, serum chromium concentrations are important, especially in infectious disease and our study will guide future work in this issue.

An acute catabolic state-like infection may influence vitamin B6 metabolism. The frequency of vitamin B6 deficiency was 35.3% in our patient population. Because there was no information about frequency of vitamin B6 deficiency in the infectious disease department in the literature, we did not compare our results with any other study. Deficiency of vitamin B6 has not been associated with any adverse clinical outcomes. Nevertheless, further investigation is needed to understand the clinical significance of vitamin B6 deficiency.

We included a small sample size and patients from a unique medical center. Therefore, confirming our findings with a larger number of subjects is warranted, and detailed analyses, including supplementation of deficient micronutrient, should be performed.

## 5. Conclusions

We revealed several features of micronutrient levels in patients who were hospitalized in the infectious disease department. Thiamine, selenium, zinc and vitamin B6 deficiencies but not chromium deficiencies are common in this clinic. New associations were found between micronutrient levels and infection type along with their adverse clinical outcomes. Hypoalbuminemia and high NRS-2002 score are major predictors of SIRS, sepsis and death. Our results provide an important basis for further nutritional attempts to improve clinical outcomes in the infectious disease department.

## Figures and Tables

**Table 1:** The basic characteristics of the patients *.

Variables	All Patients n:68	Male Patients (n:37)	Female Patients (n:31)
Age (year), mean ± SD	62.8 ± 17.5	62 ± 15.9	63.8 ± 19.4
Weight at admission (kg), mean ± SD	69 ± 13.8	71.4 ± 14.1	66.3 ± 13.3
Weight at discharge (kg), mean ± SD	69.5 ± 14.1	72.2 ± 14.3	66.4 ± 13.6
Height (cm), mean ± SD	167.8 ± 10.9	175.1 ± 7.9	159.2 ± 7.1
BMI (kg/m^2^), median (Q1 and Q3)	23.9 (20.9–27.7)	22.1 (20.4–25.3)	25 (22.3–29.4)
SIRS, *n* (%)	28 (41.2)	15 (40.5)	13 (41.9)
Sepsis, *n* (%)	18 (26.5)	9 (24.3)	9 (29)
Length of hospital stay (day), median (range)	10 (3–85)	8 (3–85)	10 (4–26)
Death, *n* (%)	11(16.2)	6 (16.2)	5 (16.1)
Types of infection, *n* (%)			
Urinary infections	16 (23.5)	6 (16.2)	10 (32.3)
Pneumonia	15 (22.1)	9 (24.3)	6 (19.4)
Soft tissue infections	11 (16.2)	7 (18.9)	4 (12.9)
Pneumonia plus urinary infections	5 (7.4)	3 (8.1)	2 (6.5)
Foreign body infections	5 (7.4)	1 (2.7)	4 (12.9)
Tuberculosis	4 (5.9)	3 (8.1)	1 (3.2)
Meningitis	2 (2.9)	1 (2.7)	1 (3.2)
Protozoal infections	2 (2.9)	2 (5.4)	-
Abscess	2 (2.9)	1 (2.7)	1 (3.2)
Other bacterial infections	6 (8.8)	4 (10.8)	2 (6.5)
Nutrition type during hospital stay, *n* (%)			
Oral	51 (75)	29 (78.4)	22 (71)
Parenteral	5 (7.4)	3 (8.1)	2 (6.5)
Enteral	1 (1.5)	-	1 (3.2)
Oral plus parenteral	8 (11.8)	5 (13.5)	3 (9.7)
Oral plus enteral	2 (2.9)	-	2 (6.5)
Enteral plus parenteral	1 (1.5)	-	1 (3.2)
NRS-2002 score, median (range)			
on admission	3 (1–7)	3 (1–7)	3 (1–7)
at first week	2 (1–7)	3 (1–7)	2 (1–7)
at discharge	2 (1–7)	2 (1–7)	2 (1–7)
Ceruloplasmin level (g/L), mean ± SD	0.89 ± 4.5	1.28 ± 5.8	0.3 ± 0.8
Micronutrients levels			
Zinc (µg/dL), median (Q1 and Q3)	64 (50.5–81.7)	65 (53.5–77.5)	62 (47.8–91.8)
Copper (µg/dL), mean ± SD	141.9 ± 41.9	139.9 ± 40.3	144.9 ± 44.9
Thiamine (ng/mL), median (Q1 and Q3)	39.1 (26.7–78.1)	37.7 (26.8–69.2)	42.2 (25.1–93.2)
Selenium (µg/L), median (Q1 and Q3)	49.9 (39.5–62.3)	49.3 (37.6–62.7)	50.5 (42–62.3)
Chromium (µg/L), mean ± SD	11.4 ± 6	11.7 ± 6.5	11 ± 5.5
Vitamin B6 (ng/mL), median (Q1 and Q3)	6.4 (3.1–15.5)	4.8 (2.6–11.2)	8 (4.6–27.3)
Vitamin B12 (pg/mL), median (Q1 and Q3)	260 (151.5–469.5)	249 (142–419)	304 (174–608)

* Significant differences were found between male and female patients for height (*p* < 0.001), BMI (*p* = 0.010), vitamin B6 (*p* = 0.046). BMI: Body mass index; SIRS: Systemic inflammatory response syndrome; NRS-2002: Nutritional Risk Screening 2002; SD: standard deviation; Q1: The lower quartiles; Q3: The upper quartiles. Normal range of micronutrients: Zinc = 70 to 150 µg/dL, Copper = 50 to 155 µg/dL, Thiamine = 33 to 99 ng/mL, Selenium = 46 to143 µg/mL, Chromium = 0.7 to 28 µg/mL, Vitamin B6 = 4.1 to 43.7 ng/mL, Vitamin B12 = 126 to 505 pg/mL; Normal range of ceruloplasmin = 0.2 to 0.6 g/L.

**Table 2:** Comparison of the micronutrients and nutritional characteristics of patients divided into groups according to SIRS, sepsis and death.

Variables	SIRS		*p*	Sepsis		*p*	Death		*p*
	Positive Negative (n:28) (n:40)			Positive Negative (n:18) (n:50)			Positive Negative (n:11) (n:57)		
Thiamine (ng/mL)	37.8 (19.6–66.5)	42.2 (30.1–95.7)	0.044	28.3 (18.7–58.3)	42.2 (29.6–88.9)	0.032	29.6 (24.1–53.3)	42 (27.7–81.6)	0.247
Vitamin B6 (ng/mL)	6.1 (2.3–17.4)	6.4 (3.5–14.8)	0.596	8.6 (2.1–21.7)	6 (3.5–14.1)	0.813	10.6 (2.2–21.1)	5.8 (3.1–15.4)	0.696
Zinc (µg/dL)	59 (47.2–67)	69 (53.5–91.7	0.042	59 (46–69.2)	65.5 (52.7–88.7)	0.131	62 (47–78)	65 (51–84)	0.681
Copper (µg/dL)	140 (114.7–171.2)	141 (110–158)	0.699	137.2 ± 48.4	143.9 ± 39.2	0.577	131.3 ± 58.2	144.3 ± 37.6	0.493
Selenium (µg/L)	45.6 (36.9–60.4)	52.6 (42.4–63.6)	0.229	49.8 ± 14.6	51.9 ± 13.9	0.596	48.1 ± 11.7	51.9 ± 14.4	0.408
Vitamin B12 (pg/mL)	318 (191.2–533.5)	218 (134.2–347)	0.144	409.5 (145.7–650)	235.5 (160.5–376.5)	0.124	421 (127–609)	228 (157–466.5)	0.237
Chromium (µg/L)	12.4 ± 5	10.6 ± 6.5	0.193	14.4 (11.4–16.4)	8.5 (5.1–16.4)	0.027	14.3 ± 4.5	10.8 ± 6.1	0.043
Multiple micronutrients deficiency	13 (46.4)	9 (22.5)	0.07	8 (44.4)	14 (28)	0.325	5 (45.5)	17 (29.8)	0.508
Cu/Zn ratio	2.54 ± 1.00	2.06 ± 0.85	0.051	2.42 ± 0.93	2.23 ± 0.96	0.471	2.18 ± 0.99	2.31 ± 0.94	0.694
NRS-2002 score on admission	5 (3.25–6)	2 (2–3)	<0.001	5 (4–6)	3 (2–4)	<0.001	5 (5–7)	3 (2–4)	<0.001
Albumin	2.86 ± 0.56	3.78 ± 0.53	<0.001	2.69 ± 0.51	3.66 ± 0.58	<0.001	2.52 ± 0.48	3.57 ± 0.61	<0.001
BMI	23.2 (20.9–27.3)	23.9 (20.8–27.8)	0.861	23.2 (21.2–26.5)	23.9 (20.6–27.7)	0.868	24 (21.3–25.6)	23.9 (20.8–27.8)	0.920

SIRS: Systemic inflammatory response syndrome, NRS-2002: Nutritional risk screening-2002, Cu: copper, Zn: Zinc, BMI: body mass index. Data are expressed as the mean ± SD, median (including the lower and upper quartiles) or noun (percentage).

**Table 3:** Results of univariate and multiple logistic regression analysis for risk factors for SIRS.

Risk Factors	OR	95% CI	*p*
**Univariate analysis**			
Thiamine level	0.98	0.97–1.00	0.074
Zinc level	0.98	0.95–1.00	0.067
Chromium level	1.05	0.97–1.14	0.211
Selenium level	0.98	0.94–1.01	0.323
Multiple micronutrients deficiency	2.98	1.04–8.53	0.041
NRS-2002 score on admission	3.56	1.97–6.43	<0.001
Age	1.03	1.005–1.07	0.022
CRP	1.01	1.004–1.019	0.002
BMI	0.97	0.87–1.08	0.589
Gender	0.94	0.35–2.48	0.907
Glucose	1.01	1.004–1.019	0.004
Urea	1.02	1.004–1.04	0.016
Leukocyte count	1.00	1.00–1.00	0.002
Hemoglobin	0.79	0.62–1.01	0.064
Albumin	0.06	0.01–0.21	<0.001
LDH	1.00	0.99–1.007	0.183
**Multiple analysis**			
Albumin	0.02	0.002–0.260	0.002
NRS-2002 on admission	2.74	1.23–6.08	0.013
Glucose	1.01	1.003–1.03	0.018

SIRS: Systemic inflammatory response syndrome; OR: odds ratio; CI: confidence interval; NRS-2002: Nutritional risk screening-2002; CRP: C reactive protein; BMI: body mass index; LDH: lactate dehydrogenase.

**Table 4:** Results of univariate and multiple logistic regression analysis for risk factors for sepsis.

Risk Factors	OR	95% CI	*p*
**Univariate analysis**			
Thiamine	0.98	0.97–1.004	0.157
Zinc	0.98	0.95–1.006	0.145
Chromium	1.09	1.00–1.20	0.051
Selenium	0.98	0.95–1.02	0.590
Multiple micronutrients deficiency	0.48	0.15–1.48	0.205
NRS-2002 score on admission	3.40	1.89–6.13	<0.001
Age	1.04	1.009–1.08	0.015
CRP	1.00	1.001–1.01	0.022
BMI	0.99	0.88–1.11	0.924
Gender	0.78	0.26–2.31	0.662
Glucose	1.00	0.99–1.009	0.330
Urea	1.02	1.008–1.04	0.005
Leukocyte count	1.00	1.00–1.00	0.043
Hemoglobin	0.68	0.51–0.91	0.010
Albumin	0.05	0.01–0.23	<0.001
LDH	1.00	1.00–1.002	0.199
**Multiple analysis**			
Albumin	0.07	0.01–0.44	0.004
NRS-2002 on admission	2.92	1.43–5.97	0.003

OR: odds ratio; CI: confidence interval; NRS-2002: Nutritional risk screening-2002; CRP: C reactive protein; BMI: body mass index; LDH: lactate dehydrogenase.

**Table 5:** Risk factors associated with hospital death according to univariate and multiple Cox’s regression.

Risk Factors	HR	95% CI	*p*
**Univariate analysis**			
Age	1.03	0.99–1.07	0.095
BMI	0.95	0.83–1.10	0.543
Chromium	1.057	0.95–1.16	0.276
Thiamine	0.99	0.98–1.009	0.436
Zinc	0.99	0.96–1.01	0.425
Selenium	0.99	0.95–1.04	0.864
Multiple micronutrients deficiency	0.61	0.18–2.03	0.424
NRS-2002 score on admission	2.79	1.55–5.03	0.001
Urea	1.01	1.001–1.019	0.037
Hemoglobin	0.64	0.44–0.92	0.018
Albumin	0.139	0.04–0.45	0.001
LDH	1.004	1.000–1.008	0.034
Leukocyte count	1.00	1.000–1.000	0.622
**Multiple analysis**			
Albumin	0.133	0.02–0.86	0.035
NRS-2002 score on admission	6.105	1.49–24.95	0.012
LDH	1.004	1.001–1.008	0.011

HR, hazard ratio; CI, confidence interval; NRS-2002: Nutritional risk screening-2002; CRP: C reactive protein; BMI: body mass index; LDH: lactate dehydrogenase.
